# Crocin exerts improving effects on indomethacin-induced small intestinal ulcer by antioxidant, anti-inflammatory and anti-apoptotic mechanisms

**DOI:** 10.30466/vrf.2018.93512.2256

**Published:** 2019-12-15

**Authors:** Sadat Ghafarzadeh, Rahim Hobbenaghi, Esmaeal Tamaddonfard, Amir Abbas Farshid, Mehdi Imani

**Affiliations:** 1 *Department of Pathobiology, Faculty of Veterinary Medicine, Urmia University, Urmia, Iran;*; 2 *Department of Basic Sciences, Faculty of Veterinary Medicine, Urmia University, Urmia, Iran.*

**Keywords:** Crocin, Indomethacin, Ranitidine, Small intestinal ulcer

## Abstract

Crocin is a plant-derived carotenoid and bears potent antioxidant property. Ranitidine (a histamine H_2_ receptor blocker) is used for peptic ulcer treatment. The present study was planned to investigate the effects of crocin and ranitidine on indomethacin-induced ulcer in small intestine of rats. Animals were randomized into two major groups including indo-methacin (10.00 mg kg^-1^, ulcer group, 48 rats) and normal saline (1.00 mL kg^-1^, intact group, 48 rats) groups. Each of these two major groups was subdivided into eight subgroups for intra-peritoneal (IP) injections of normal saline, crocin (2.50, 10.00 and 40.00 mg kg^-1^), ranitidine (5.00 and 20.00 mg kg^-1^), crocin (2.50 and 10.00 mg kg^-1^) plus ranitidine (5.00 mg kg^-1^). Indomethacin induced intestinal ulcer was characterized by bleeding, inflammation, epithelial hyperplasia and crypt loss. This non-steroidal anti-inflammatory drug (NSAID), indomethacin decreased goblet cell number and superoxide dismutase (SOD) activity and increased small intestine weight, organo-somatic index (OSI), malodealdehyde (MDA), tumor necrosis factor-α (TNF-α) and caspase-3 contents of intestine. Crocin resolved all the above-mentioned parameter changes induced by indomethacin. These treatments produced no significant effects on the above-mentioned parameters of intact group. The results of the present study showed tissue protective and anti-ulcer effects of crocin on small intestine by antioxidant, anti-inflammatory and anti-apoptotic mechanisms. Ranitidine alone showed no effect; however, in combination with crocin it exerted recovery effects. It is recommended that crocin, be considered as a therapeutic agent for NSAIDs-induced intestinal damage management.

## Introduction

Non-steroidal anti-inflammatory drugs (NSAIDs) are commonly used for their ant-inflammatory, analgesic and antipyretic effects for treatment of rheumatoid arthritis, osteoarthritis, ischemic cardiovascular and cerebro-vascular diseases.^1^ However; they are associated with gastrointestinal tract adverse events. Beside effects on the mucosa of upper gastrointestinal tract, NSAIDs also damage the small intestine mucosa by producing multiple mucosal erosions, ulcers and bleeding.^2^ Gastrointestinal lesions induced by NSAIDs can be managed using alone or combination treatments with mucosal protective agents (misoprostol, rebamipide), antibiotics and probiotics, food constituents (lactoferrin), proton pump inhibitors (omeprazole and lansoprazole) and histamine H_2_ receptor antagonists such as ranitidine and cimetidine.^3^^,^^4^

*Crocus sativus* (saffron), as an herbaceous plant, possesses a number of medicinally important activities such as antihypertensive, anticonvulsant, antigenotoxic, anxiolytic, antioxidant, antidepressant, antinociceptive and anti-inflammatory effects.^5^ Carotenoids represent the main constituents of saffron and their cleavage results in formation of apocarotenoids such as crocin, picocrocin and safranal.^6^ Crocin(C_44_H_64_O_24_), with potent antioxidant and anti-inflammatory properties, exerts tissue protective effects on ischemia/reperfusion models at various body organs such as brain, heart, kidney, liver and stomach.^7^

Regarding the fact that oxidative stress plays an important role in pathophysiological mechanisms of gastro- intestinal mucosal ulcer induced by NSAIDs, natural phytochemicals with a potent antioxidant activity have been suggested for ulcermanagement.^8 ^In this context, recent studies have reported protective effects of crocin on indomethacin- and ethanol-induced gastric ulcers in rats.^9^^,^^10^ On the other hand, it is important to elucidate the effect of antisecretory drugs such as histamine H_2_ receptor antagonists on NSAID-induced intestinal lesions, because many patients take these drugs to prevent upper gastrointestinal side effects that are associated with NSAID use.^11^In experimental studies, some of these antagonists such as roxatidine and lafutidine, but not cimetidine and famotidine were found to protect mucosa against indo-methacin-induced intestinal ulceration.^12^^,^^13^ Considering the above-mentioned findings, the present study was planned to investigate the effects of separate and combined treatments with crocin and ranitidine, a histamine H_2_ receptor antagonist, on indomethacin-induced small intestine ulcer. Physical (fecal occult blood, relative organ weight measurements), pathological (macroscopic and light microscopic evaluations) and biochemical assay of small intestine were performed to clarify the possible mechanisms.

## Materials and Methods

**Animals. **Ninety-six adult male Wistar rats (200 - 220 g) were used in this study. Rats were maintained in a laboratory under controlled 12 hr light-dark cycle and ambient temperature (22.00 ± 0.50 ˚C) with *ad libitum* food and water. The Ethics Committee of the Faculty of Veterinary Medicine of Urmia University (AECVU-174-2018) approved the research and animal care procedures. 

**Chemicals.** Indomethacin, crocin and ranitidine were purchased from Sigma-Aldrich Co. (St. Louis, USA). Superoxide dismutase assay kit (Cayman chemicals, USA), tumor necrosis factor-α assay kit (Elabscience, Texas, USA) and caspase-3 assay kit (Boster Biological Technology, Pleasanton, USA) were purchased. Analytical chemicals such as thiobarbituric acid were purchased from Merck chemical Co. (Darmstadt, Germany).

**Treatment groups. **According to the intra-gastric administrations of normal saline and indomethacin, 96 rats were randomized into two normal saline (48 rats) and indomethacin (48 rats) major groups. The indomethacin major group was subdivided into eight subgroups including 10.00 mg kg^-1^ indomethacin plus normal saline (Indo 10 + Ns), 10.00 mg kg^-1^ indomethacin plus 2.50 mg kg^-1^ crocin (Indo 10 + Cro 2.5), 10 mg kg^-1^ indomethacin plus 10.00 mg kg^-1^ crocin (Indo 10 + Cro 10), 10.00 mg kg^-1^ indomethacin plus 40.00 mg kg^-1^ crocin (Indo 10 + Cro 40), 10.00 mg kg^-1^ indomethacin plus 5.00 mg kg^-1^ ranitidine (Indo 10 + Ran 5), 10.00 mg kg^-1 ^indomethacin plus 20.00 mg kg^-1^ ranitidine (Indo 10 + Ran 20), 10.00 mg kg^-1^ indomethacin plus 2.50 mg kg^-1^ crocin plus 5.00 mg kg^-1 ^(Indo 10 + Cro 2.5 + Ran 5) and 10.00 mg kg^-1^ indo-methacin plus 10.00 mg kg^-1^ crocin plus 5.00 mg kg^-1^ (Indo 10 + Cro 10 + Ran 5). The similar subgroup subdivision and treatment were done for normal saline major group, however, these subgroups received normal saline by gavage instead of indomethacin. The purpose of addition of normal saline to divided subgroups was to explore the effects of crocin and ranitidine and their combination treatments in intact (without intestinal ulcer‎) rats on respective parameters. Crocin and ranitidine were dissolved in normal saline and administered intra-peritoneally in a constant volume 1.00 mL kg^-1^ at 1, 4, and 16 hr after oral administrations of normal saline and indomethacin. The used doses of crocin and ranitidine were in accordance to previous studies in which crocin (5.00 - 40.00 mg kg^-1^) and ranitidine (3.00 - 30.00 mg kg^-1^) were used.^14^^,^^15^

**Induction of intestinal ulcer.** We used indomethacin for induction of intestinal ulceration. A suspension of indomethacin in normal saline was provided and at a dose of 10.00 mg kg^-1^ in a constant volume 1.00 mL kg^-1^ was administered using intra-gastric tube (gavage) in non-fasted rats.^16^ Animals of normal saline major group were treated identically, however, they received normal saline with same liquid measure by gavage.

**Fecal occult blood test.** Twenty-two hours after induction of intestinal ulcer, each rat was placed in plexiglass chamber as a new environment for a period of 15 min.^17^ At the end of this period, 4-6 fresh fecal pellets were collected for achieving fecal occult blood test. A small fecal sample was smeared on guaiac paper and three drops of hydrogen peroxide was applied on the sample.^18^ Appearance of blue color within 30 sec was considered as a positive response. Positive and negative responses were expressed as percentage by the following formula: 

Number of positive or negative responses/Total number of fecal samples × 100. 

**Quantification of intestinal ulcer.** Twenty-four hours after normal saline and indomethacin administration, the rats were euthanized by deep ether anesthesia. The small intestine was removed and opened along the anti-mesenteric attachment. Thereafter, the mucosal surface was washed using cooling normal saline, derided and weighted. The number of mucosal ulcers including spot, circular longitudinal ulcers was counted as described previously.^19^ In addition, organo-somatic index (OSI) was calculated according the following formula:^20^

*OSI = [Small intestine weight (g)/Body weight (g)]* *×* *100*

This index reflects the relative organ weight caused by a respective organ weight to body weight changes.^21^

**Tissue collection. **Immediately after counting intestinal mucosal ulcers, distal portion of small intestine was separated from each small intestine specimen and divided into two halves, one half for histopathological and another for biochemical evaluations. For histo-pathological evaluation, jejunum and ileum segments were fixed in 10.00% buffer formal saline, and for biochemical assay, the specimens were rinsed in ice-cold saline solution. It has been reported that indomethacin produces more mucosal ulceration in the distal portion of jejunum and ileum in rats.^22^

**Microscopic scoring. **The formalin fixed tissues were dehydrated and embedded in paraffin and cut into 5.00 µm sections. Sections were hydrated and stained with Hematoxylin and Eosin (H & E). The microscopic scoring was performed for inflammatory cell infiltration severity (1; minimal: < 10.00%, 2; mild: 10.00 - 25.00%, 3: moderate: 26.00 - 50.00%, 4; marked: > 51.00%) and extent (1: mucosal, 2: mucosal and submucosal, 3: mucosal, submucosal and transmural), epithelial changes including hyperplasia (1; minimal: < 25.00%, 2 or 3: mild: 25.00 - 35.00%, 4 or 5; marked: > 51.00%), goblet cell loss (1 or 2; minimal: < 20.00%, 2 or 3; mild: 21.00 - 35.00%, 3 or 4; moderate: 36.00 - 50.00%, 4; marked: > 50.00%) and erosion (1 - 4; loss of surface epithelium) and mucosal architecture such as villous blunting (1 to 3; mild, 2 to 4; moderate, 3 to 5; villous atrophy) as previously described by Erben *et al*.^23^

**Biochemical assay. **Small intestine tissue segments were cut into small pieces and homogenized at 4.00˚C in 2.00mLof ice-cold saline with glass homogenizer. The tissue MDA level was measured spectrophotometrically (UV-975; Jasco, Tokyo, Japan) by the thiobarbituric (TBA) acid method,^20^ and expressed as nmol per mg of protein. Superoxide dismutase (SOD) activity of small intestine tissue was determined by superoxide dismutase assay kit according to the manufacture instruction (Cayman Chemical, Ann Arbor, USA). Small intestine tissue SOD activity was expressed as U per mg protein. Small intestine tissue content of TNF-αwas measured by ELISA according to the kit instruction (Bioscience, Santa Clara, USA). The TNF-α content of small intestine tissue was expressed as pg per mg of protein. Caspase-3 level in small intestine tissue was determined using ELISA assay according to the kit instruction (Elabscience Biotechnology Co. Ltd., Wuhan, China), and expressed as ng per mg protein. Small intestine tissue protein concentration was measured using Bradford protein assay.^24^

**Statistical analysis.** Statistical comparisons were performed using the GraphPad Prism (version 5.0; GraphPad software, San Diego, USA). Significance of fecal occult blood, intestinal weight, OSI, ulcer number and biochemical data were assessed by one-way (ANOVA) followed by Tukay’s post hoc test. Because of semi-quantitative nature of data obtained from microscopic alterations, Kruskal-Wallis and post hoc Dunn’s multiple comparison tests were applied. The significant level was set at *p *< 0.05.

## Results

Normal saline, crocin, ranitidine and crocin plus ranitidine treatments in the normal saline (intact) subgroup showed no significant effects on physical, pathological and biochemical parameters (*p *> 0.05), (data not shown).

Indomethacin induced fecal occult blood, and with no effect on body weight, increased small intestine weight and OSI and produced intestinal ulceration. Crocin (2.50 mg kg^-1^), ranitidine (5.00 and 20.00 mg kg^-1^) and crocin (2.50 mg kg^-1^) plus ranitidine (5.00 mg kg^-1^) produced no significant effects, whereas 10.00 and 40.00 mg kg^-1^ crocin and a combination of crocin (10.00 mg kg^-1^) with ranitidine (5.00 mg kg^-1^) significantly restored fecal occult blood and decreased the increased small intestine weight, OSI and the number of intestinal ulcers (*p *< 0.05). A significant difference was observed between combination treatments (*p *< 0.05; Table 1).

**Fig. 1:**
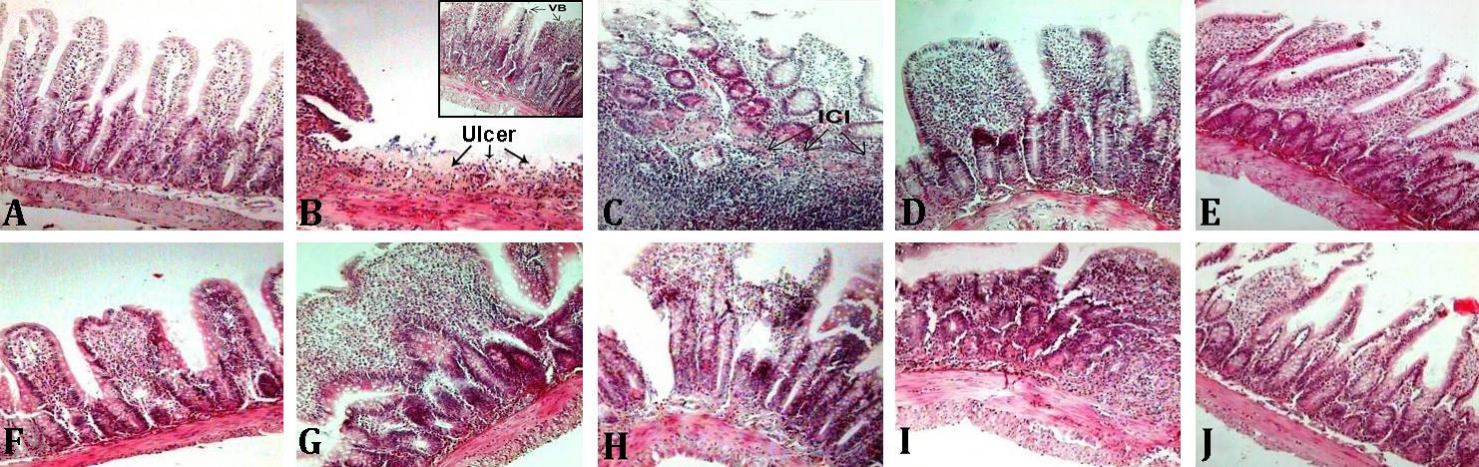
Photomicrographs of small tissue sections of experimental groups. **A) **Intact (Ns + Ns): shows the normal architecture; **B** and **C) **Indo (10) + Ns: shows ulcer (arrows), inflammatory cell infiltration (ICI; arrows), crypt destruction and villous blunting (VB; arrows in inset); **D) **Indo (10) + Cro (2.5): no recovery effect is seen; **E) **Indo (10) + Cro (10): moderate recovery especially in leucocyte infiltration is seen; **F)** Indo (10) + Cro (40): a marked recovery is seen; **G) **Indo (10) + Ran (5): no recovery effect is seen; **H) **Indo (10) + Ran (20): no recovery effect is seen; **I) **Indo (10) + Cro (2.5)+Ran (5): no recovery effect is seen; and **J) **Indo (10) + Cro (10) + Ran (5): a moderate recovery effect is seen, (H & E, 100×).The numbers inside parenthesis reflect drug doses as mg kg^-1^. Ns: normal saline, Indo: indomethacin, Cro: crocin, Ran: ranitidine

**Fig. 2:**
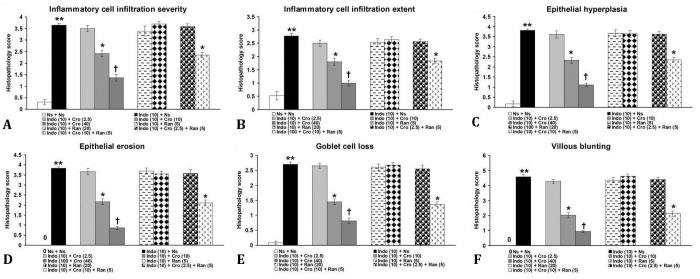
Effects of separate and combination treatments with crocin and ranitidine on **A)** Inflammatory cell infiltration severity; B) Inflammatory cell infiltration extent; C) Epithelial hyperplasia; D) Epithelial erosion; E) Goblet cell loss; and F) Villous blunting of small intestinal mucosa induced by indomethacin. Data are the mean ± SEM from six rats in each group. The numbers inside parenthesis reflect drug doses as mg kg^-1^.** *p* < 0.001 compared with Ns + Ns group, * *p* < 0.05 compared with Indo + Ns group, ^†^*p* < 0.05 compared with Indo + Ns group. Ns: normal saline, Indo: indomethacin, Cro: crocin, Ran: ranitidine

Control small intestine had normal architecture (Fig. 1A) with normal histopathological scores (Figs. 2A-2F). Indomethacin produced inflammatory cell infiltration, epithelial changes and villous blunting (Figs. 1B, 1C and Figs. 2A-2F). 

**Table 1:** Effects of crocin, ranitidine and their combination on fecal occult blood, body weight, intestinal weight, organo-somatic index (OSI) changes and number of small intestine ulcers induced by indomethacin in rats (mean ± SEM)

**Groups**	**Fecal occult blood (%)**	**Body weight (g)**	**Small intestine weight (g)**	**Organo-somatic index**	**Number of small intestine ulcers**
**Ns + Ns (control)**	0.00 ± 0.00^a^	210.2 ± 4.73^a^	2.65 ± 0.31^a^	2.69 ± 0.09^a^	0.00 ± 0.00^a^
**Indo (10) + Ns**	97.2 ± 2.78^b^	207.9 ± 3.78^a^	7.52 ± 0.27^b^	3.61 ± 0.08^b^	89.3 ± 5.1^b^
**Indo (10) + Cro (2.5)**	88.9 ± 5.55^b^	207.5 ± 2.91^a^	7.18 ± 0.25^b^	3.47 ± 0.14^b^	79.8 ± 4.54^b^
**Indo (10) + Cro (10)**	55.6 ± 7.03^c^	209.7 ± 3.51^a^	6.51 ± 0.15^c^	3.11 ± 0.07^c^	57.3 ± 4.41^c^
**Indo (10) + Cro (40)**	25.1 ± 3.71^d^	212.1 ± 3.95^a^	5.92 ± 0.16^c^	2.79 ± 0.11^c^	32.1 ± 3.69^d^
**Indo (10) + Ran (5)**	83.3 ± 8.61^b^	210.5 ± 4.12^a^	7.22 ± 0.22^b^	3.43 ± 0.09^b^	75.7 ± 7.74^b^
**Indo (10) + Ran (20)**	80.6 ± 7.95^b^	208.9 ± 3.69^a^	6.83 ± 0.29^b^	3.28 ± 0.16^b^	81.2 ± 6.01^b^
**Indo (10) + Cro (2.5) + Ran (5)**	86.1 ± 5.12^b^	210.4 ± 4.58^a^	6.93 ± 0.25^b^	3.29 ± 0.12^b^	78.8 ± 5.67^b^
**Indo (10) + Cro (10) + Ran (5)**	44.5 ± 5.54^c^	208.4 ± 4.56^a^	6.31 ± 0.14^c^	3.03 ± 0.11^c^	48.8 ± 3.34^c^

Ns: normal saline, Indo: indomethacin, Cro: crocin, Ran: ranitidine. The numbers inside the parenthesis represent the used chemical compound doses as mg kg^-1^. Different superscript letters indicate significant differences at *p *< 0.05.

Crocin (2.50 mg kg^-1^, Fig. 1C and Figs. 2A-2F), ranitidine (5.00 and 10.00 mg kg^-1^, Figs. 1F, 1G and Figs. 2A-2F), and a combination treatment with 2.50 mg kg^-1^ crocin plus 5.00 mg kg^-1^ ranitidine (Fig. 1H and Figs. 2A-2F) produced no significant effects on histopathological changes induced by indomethacin (*p *> 0.05). Crocin at doses of 10.00 mg kg^-1^ (Fig. 1D and Figs. 2A-2F) and a combination of 10.00 mg kg^-1^ crocin and 5.00 mg kg^-1^ ranitidine (Fig. 1I and Figs. 2A-2F) significantly improved indomethacin-induced small intestine damages and histopathology scores (*p *< 0.05). Crocin at a dose of 40.00 mg kg^-1^ (Fig. 1E and Figs. 2A-2F) produced more significant improving effects (*p *< 0.01). Significant differences were observed between combination treatments (*p *< 0.05; Figs. 1H, 1I and Figs. 2A-2F). 

Table 2 shows the small intestine tissue biochemical parameters. Indomethacin significantly increased MDA, TNF-α and caspase-3 contents (*p *< 0.01) and significantly decreased SOD activity in the small intestine tissue (*p *< 0.05). Crocin (2.50, 10.00 and 40.00 mg kg^-1^) and a combination of crocin (10.00 mg kg^-1^) and ranitidine (5.00 mg kg^-1^) significantly restored the increased levels of MDA, TNF-α, caspase-3 as well as the decreased activity of SOD (*p *< 0.05). The increased levels of MDA, TNF-α, caspase-3 and the decreased activity of SOD induced by indomethacin were not changed by 5.00 and 20.00 mg kg^-1^ ranitidine and combination of 2.50 mg kg^-1^ crocin with 5.00 mg kg^-1^ ranitidine. There were significant differences between the effects of combination treatments on biochemical changes (*p *< 0.05; Table 2).

**Table 2:** Effects of crocin, ranitidine and their combination on the changes in small intestinal tissue MDA, TNF-α and caspase-3 levels and SOD activity induced by indomethacin in rats (mean ± SEM)

Groups	Malondialdehyde (nmol per mg protein)	Tumor necrosis factor-α (pg per mg protein)	Caspase-3 (ng per mg protein)	Superoxide dismutase (U per mg protein)
**Ns + Ns (control)**	3.75 ± 0.16^a^	10.89 ± 0.45^a^	2.24 ± 0.17^a^	7.49 ± 0.29^a^
**Indo (10) + Ns**	7.75 ± 0.55^b^	42.51 ± 1.92^b^	5.19 ± 0.39^b^	2.94 ± 0.22^b^
**Indo (10) + Cro (2.5)**	6.05 ± 0.21^c^	32.88 ± 1.17^c^	4.09 ± 0.36^c^	3.76 ± 0.16^c^
**Indo (10) + Cro (10)**	4.31 ± 0.22^d^	24.76 ± 1.68^d^	3.39 ± 0.19^d^	5.48 ± 0.17^d^
**Indo (10) + Cro (40)**	3.16 ± 0.19^e^	17.18 ± 2.06^e^	2.32 ± 0.15^e^	7.69 ± 0.18^e^
**Indo (10) + Ran (5)**	7.03 ± 0.33^b^	39.43 ± 2.08^b^	4.93 ± 0.17^b^	3.06 ± 0.24^b^
**Indo (10) + Ran (20)**	7.19 ± 0.23^b^	42.29 ± 3.04^b^	4.87 ± 0.31^b^	3.09 ± 0.19^b^
**Indo (10) + Cro (2.5) + Ran (5)**	6.86 ± 0.27^b^	40.51 ± 2.74^b^	4.63 ± 0.23^b^	3.01 ± 0.29^b^
**Indo (10) + Cro (10) + Ran (5)**	3.84 ± 0.22^d^	27.35 ± 2.87^d^	3.13 ± 0.18^d^	5.28 ± 0.21^d^

Ns: normal saline, Indo: indomethacin, Cro: crocin, Ran: ranitidine. The numbers inside the parenthesis represent the used chemical compound doses as mg kg^-1^. Different superscript letters indicate significant differences at *p *< 0.05.

## Discussion

In the present study, we confirmed indomethacin-induced intestinal bleeding by fecal occult blood (FOB) test. The FOB is a sign of gastrointestinal diseases such as intestinal ulcers and colorectal cancer and FOB test is a simple, quick and economical method to detect FOB in experimentally-induced intestinal ulcers, for example NSAIDs- small intestinal damages.^25^^,^^26^ Indomethacin can cause damage by changing the hydrophobic nature of the intestinal mucosa and increasing permeability leads to bleeding of the small intestine.^27^ Our results showed that indomethacin increased small intestinal weight and OSI. The increased small intestine weight may be related to mucosal, submucosal or muscularis hyperemia and edema combined with inflammatory cell infiltration and the associated exudate.^28^ Body weight was not changed in our study, so the increased OSI might be related to increase of small intestine weight. In the present study, indomethacin produced numerous ulcers in the mucosa of small intestine. Indomethacin-treated rats were found to develop pointed (< 5.00 mm) and longitudinal (> 5.00 mm) ulcers scattered throughout the small intestine with increasing in the number from the proximal to the end of the small intestine.^19^ In our study, histopathological evaluation of small intestine sections showed extensive inflammatory cell infiltration, epithelial hyperplasia, epithelial erosion, goblet cell loss and villous blunting in indomethacin-treated rats. It has been reported that indomethacin causes an inflammatory reaction characterized by epithelial losses, ulcers, inflammatory cell infiltration into the lamina propria, submucosa and serosa and shortening of crypts.^16^ The proposed mechanisms underlying NSAID-induced intestinal histopathological changes include reduced epithelial anion and mucus secretion, hypermotility, reduced blood flow, increased inflammatory cell infiltration, and bacterial trans-location.^29^ Final step of our present results showed that indomethacin increased MDA, TNF-α and caspase-3 levels, and decreased SOD activity in small intestine tissue. Cyclooxygenase pathways 1 and 2 (COX1 and COX2), oxidative stress, cytokines and apoptosis play important roles in the pathophysiology of NSAIDs-induced entero-pathy.^29^ In this context, indomethacin increased lipid peroxidation in the ileum of mice and decreased SOD activity in the small intestine of rats.^16^^,^^30^ In addition, this NSAID elevated TNF-α production and increased caspase-3 expression in small intestinal of mice.^31^

Our present study demonstrated that crocin reduced intestinal bleeding and decreased the increased intestinal weight and OSI. These effects might be associated with anti-hemorrhagic, anti-edematous and organ weight loss inhibiting properties of crocin. In acetic acid-induced ulcerative colitis, body and colon weight loss and colon tissue hemorrhages and interstitial edema were attenuated by crocin treatment in rats.^20^ Crocin bears a potent controlling effect on blood vessel endothelial cell function for inhibiting vascular permeability.^32^ Thepresent results showed that crocin reduced the number of intestinal ulcers induced by indomethacin. There are no reports showing the effects of crocin on small intestine ulcer induced by indomethacin. However, the increased gastric ulcer index induced by ethanol and indomethacin were attenuated by crocin treatment in rats.^9^^,^^10^ Moreover, crocin treatment ameliorated acetic acid-induced ulcerative colitis in rats.^20^ In the present study, crocin improved indomethacin-induced small intestine histo-pathological changes including extensive inflammation, epithelial changes and villous blunting. Crocin recovered mucosal layer destruction, submucosal edema, extensive leukocyte infiltration and crypt destruction in acetic acid induced ulcerative colitis in rats.^20^ Moreover, acrylamide-induced histopathological changes including villous shortening and degeneration, surface epithelium and crypt degeneration in the small and large intestines were recovered by crocin.^33^ The present study showed restoration effects of crocin on the increased levels of MDA, TNF-α and caspase-3 and the decreased activity of SOD in small intestine. It has been reported that crocin exerted improving effects on the increased contents of MDA, TNF-α and increased the decreased activity SOD of colon tissue in acetic-acid- induced ulcerative colitis.^20^ In addition, crocin inhibited oxidative stress and stimulated antioxidant enzyme production in acrylamide-induced small and large intestine damages.^33^ Crocin protected rat gastric mucosa against ethanol-induced injury via anti-inflammatory, anti-oxidative, anti-apoptotic and mucin-secretagogue mechanisms.^9^ In this context, crocin possessed gastro-protective effects against indomethacin-induced gastric ulcers by decreasing the increased expression of caspase-3 as well as the elevated level of MDA in rats.^10^ The above-mentioned findings and the results of the present study indicated that crocin could produce protective effects on indomethacin-induced small intestine damage by anti-bleeding, anti-edematous, anti-oxidant, anti-inflammatory and anti-apoptotic mechanisms. 

The present study could not show a protective effect of ranitidine on small intestine ulcer induced by indomethacin. This is in agreement with other findings in which histamine H_2_ receptor antagonists such as cimetidine and famotidine exerted no protective effects on indomethacin-induced small intestine damage.^11^^,^^12^ Interestingly, Satoh *et al.* reported that cimetidine, ranitidine and famotidine augmented the increase of intestinal damage caused by indomethacin.^15^ Although the exacerbation effect mechanisms of histamine H_2_ receptor antagonists on NSAIDs-induced intestinal lesions are not fully understood, intestinal MDA level elevation especially by ranitidine, goblet cell loss (mucus depletion), increase of intestinal motility, long-term intestinal lumen pH changes and subsequent dysbiosis have been suggested.^15^^,^^34^ In contrast to these findings, other histamine H_2_ receptor antagonists such as lafutidine and roxatidine was found to possess protective effect against intestinal damages induced by subcutaneous (SC) injection of indomethacin in rats.^11^^,^^12^ These discrepancies may be related to kind of antagonist and route of administration. Lafutidine and roxatidine belongs to second-generation histamine H_2_ receptor antagonists with fewer side effects, whereas cimetidine, ranitidine and famotidine comprise the first-generation.^35^ However, further studies would be required to shed more lights on the management of NSAIDs-induced intestinal ulcer by histamine H_2_ receptor antagonists.

The results of the present study demonstrated that a combination treatment with low doses of crocin (2.50 mg kg^-1^) and ranitidine (5.00 mg kg^-1^) did not affect indomethacin-induced intestinal damage, whereas by increasing the dose of crocin to 10.00 mg kg^-1^, protective effects form combination treatment were observed. This indicated that concomitant use of a protective agent such as an antioxidant not only improved NSAIDs-induced intestinal damage, but also could prevent ulcer exacerbation. In this context, it has been found that mucosal protective agents including misoprostol, irsogladine, rebamipide and mucin prevented the exacerbation of diclofenac (NSAID)-induced small intestine lesions by antisecretory drugs such as ranitidine.^15^ Co-administration of quercetin, a potent antioxidant, and ranitidine protected small intestinal mucosa by preventing exacerbation effect of ranitidine on diclofenac-induced lesion as well as reducing intestinal tissue level of MDA.^35^ Curcumin, a constituent of turmeric with a potent antioxidant activity, completely prevented exacerbation effect of pantoprazole (an antisecretory agent) on diclofenac-induced small intestine ulcer in rats.^36^

In conclusion, the results of the present study demonstrated that indomethacin through activation of oxidative stress, inflammatory cytokine production and apoptotic stimulation provoked small intestine ulcer supported by bleeding, macroscopic and microscopic outcomes. Crocin, but not ranitidine, protected small intestinal mucosa by anti-oxidant, anti-inflammatory and anti-apoptotic mechanisms. Co-administration of crocin with ranitidine also produced a protective effect. The use of crocin alone, and in combination with antisecretory agent could be considered as a new therapeutic agent in NSAIDs-induced enteropathy management.
